# Laser Capture Microdissection and Multiplex-Tandem PCR Analysis of Proximal Tubular Epithelial Cell Signaling in Human Kidney Disease

**DOI:** 10.1371/journal.pone.0087345

**Published:** 2014-01-27

**Authors:** Ray Wilkinson, Xiangju Wang, Andrew J. Kassianos, Steven Zuryn, Kathrein E. Roper, Andrew Osborne, Sandeep Sampangi, Leo Francis, Vishwas Raghunath, Helen Healy

**Affiliations:** 1 Conjoint Kidney Research Laboratory, Pathology Queensland, Brisbane, Queensland, Australia; 2 Department of Renal Medicine, Royal Brisbane and Women's Hospital, Brisbane, Queensland, Australia; 3 Pathology Queensland, Brisbane, Queensland, Australia; 4 Institute of Health and Biomedical Innovation, Queensland University of Technology, Brisbane, Queensland, Australia; 5 Medical School, University of Queensland, Brisbane, Queensland, Australia; Biomedical Research Foundation of the Academy of Athens, Greece

## Abstract

Interstitial fibrosis, a histological process common to many kidney diseases, is the precursor state to end stage kidney disease, a devastating and costly outcome for the patient and the health system. Fibrosis is historically associated with chronic kidney disease (CKD) but emerging evidence is now linking many forms of acute kidney disease (AKD) with the development of CKD. Indeed, we and others have observed at least some degree of fibrosis in up to 50% of clinically defined cases of AKD. Epithelial cells of the proximal tubule (PTEC) are central in the development of kidney interstitial fibrosis. We combine the novel techniques of laser capture microdissection and multiplex-tandem PCR to identify and quantitate “real time” gene transcription profiles of purified PTEC isolated from human kidney biopsies that describe signaling pathways associated with this pathological fibrotic process. Our results: (i) confirm previous in-vitro and animal model studies; kidney injury molecule-1 is up-regulated in patients with acute tubular injury, inflammation, neutrophil infiltration and a range of chronic disease diagnoses, (ii) provide data to inform treatment; complement component 3 expression correlates with inflammation and acute tubular injury, (iii) identify potential new biomarkers; proline 4-hydroxylase transcription is down-regulated and vimentin is up-regulated across kidney diseases, (iv) describe previously unrecognized feedback mechanisms within PTEC; Smad-3 is down-regulated in many kidney diseases suggesting a possible negative feedback loop for TGF-β in the disease state, whilst tight junction protein-1 is up-regulated in many kidney diseases, suggesting feedback interactions with vimentin expression. These data demonstrate that the combined techniques of laser capture microdissection and multiplex-tandem PCR have the power to study molecular signaling within single cell populations derived from clinically sourced tissue.

## Introduction

It is widely acknowledged that the epithelial cells of the proximal tubule (PTEC) play a central role in interstitial fibrosis, following cellular insults such as excessive protein exposure and oxidative stress [1]–[5]. These insults lead to perturbation of the complex interactions of growth factors, cytokines and chemokines which maintain the homeostasis of these cells, leading to the over-expression of genes involved in inflammatory responses [6]–[8] and epithelial to mesenchymal transition (EMT) [9]–[12], both pathobiological processes resulting in a fibrotic phenotype. However, research implicating PTEC involvement in interstitial fibrosis comes predominantly from animal models or in-vitro studies using transformed cell lines or primary PTEC cultures. The relevance of translating PTEC knowledge derived from these models to human disease has not been established. Attempts to elucidate transcription profiles in human kidney disease tissue have generally taken a global approach, analyzing gene expression from whole kidney biopsies [13], [14] or tissue sections with mixed cellular populations [15].

In this work we exploit the recent developments in microscopic dissection using Laser Capture Microdissection (LCM) to target the single cell PTEC population ex-vivo. The technology allows the visualisation of proximal tubules within renal biopsies from patients with kidney disease and the subsequent capture/isolation of PTEC from these tubules using laser catapult energy [16]. This technology has been recently used in human kidney research of gene expression in the glomerulus [17]–[19], to study one or two genes across different compartments of the kidney [20], [21] or to analyse PTEC collected from proteinuric disease biopsies using gene microarray technology [22], [23]. Gene microarray analysis studies provide vast amounts of transcriptional data but have a number of inherent difficulties including: (i) difficult and expensive to undertake, (ii) require duplicate samples to eliminate artifactual data, (iii) take weeks or months to analyse the large volumes of data and (iv) require large amounts of input RNA or, when not available as occurs in the clinical setting, non-specific pre-amplification of high quality RNA.

To remove bias, commonly encountered when non-specifically pre-amplifying the picogram quantities of RNA typically obtained from human biopsies using LCM, we utilised targeted gene transcription analysis using primer-specific nested amplification. This methodology, known as multiplex tandem-PCR (MT-PCR), has been reported to amplify cDNA from as little as 10 picograms of RNA and was designed to amplify degraded RNA recovered from formalin-fixed paraffin embedded tissue [24]. Here we use MT-PCR analysis of LCM-isolated PTEC from clinical human kidney biopsies to provide transcriptional data on the four major pathobiological pathways associated with kidney fibrosis: (i) inflammation, (ii) collagen deposition, (iii) EMT and (iv) apoptosis. We relate changes in the transcriptional profiles of the pathways to the clinical phenotype of the patients. These technologies are readily translatable to human diagnostics of the future where the prognostic power of PTEC may direct therapies to specific targets.

## Methods

### Patient and Tissue sample details

Control tissues were obtained from the healthy portions of malignant nephrectomies (n = 8) and were validated as normal at the macroscopic and microscopic level by the consulting renal pathologist. Diseased samples (n = 96) were collected from native kidney biopsy material that were surplus to diagnostic requirements. Biopsies were frozen in OCT within 20 minutes of collection and stored at −80°C prior to processing. When biopsies/patients were grouped according to the clinical diagnosis of acute or chronic disease, defined clinically prior to histopathological diagnosis, we had a total of 26 acute, 16 chronic, 51 acute-on-chronic and 3 undefined. Final disease diagnoses, of which there were three or more patients per disease group (n = 87), included IgA nephropathy (IgAN; n = 14), focal segmental glomerulosclerosis (FSGS; n = 11), pauci-immune glomerulonephritis (PI; n = 11), membranous nephropathy (MN; n = 9), interstitial nephritis (IN; n = 7), protein deposition disease (PDD; n = 7), minimal change disease (MCD; n = 6), diabetic nephropathy (DN; n = 6), lupus nephritis (LN; n = 5), membranoproliferative glomerulonephritis (MPGN; n = 4), thrombotic microangiopathy (TMA; n = 4) and acute tubular necrosis (ATN; n = 3). The renal pathologist also scored the histological grading of the interstitium of all disease biopsies using a scale modified from the Banff system [25]. Briefly, overall cortical inflammation (OCI), unscarred cortical inflammation (UCI) and interstitial fibrosis/tubular atrophy (IF/TA) were each graded on a scale of 0–4 (0 =  not present, 1 =  up to 5%, 2 = 6–25%, 3 = 26–50% and 4 =  greater than 50%) and yes/no for the presence of acute tubular injury (ATI), neutrophils and eosinophils. This grading was based upon the analysis of twenty individual 4 µm sections from each biopsy, which had been stained with haematoxylin and eosin or Masson's trichrome. Kidney function (estimated glomerular filtration rate (eGFR)) was calculated using the MDRD method by AUSLAB (Queensland Health) and proteinuria was described by urine protein to creatinine ratio (uPCR).

### Ethics statement

All disease biopsy samples were used following written informed patient consent under human ethics approval from the Royal Brisbane and Women's Hospital (RBWH) (2002/011) and the Queensland Institute of Medical Research (QIMR) (P293).

### LCM

10 µm thick cryosections were cut under RNase-free conditions and mounted on MembraneSlide 1.0 PEN slides (Carl Zeiss, Munich, Germany) treated with 20 min of UV exposure prior to use. Cryosections were stored at −80°C for a maximum of 48 hours or processed immediately for staining of endogenous alkaline phosphatase, exclusively expressed by PTEC in kidney cortex, by a one minute fixation in 70% ethanol followed by staining with SigmaFast BCIP/NBT (Sigma-Aldrich, St Louis, MO, USA) for 5 min. Cryosections were then dehydrated for 1 min each in 70%, 90% and 100% ethanol and PTEC were collected from these sections for a maximum time of 30 min, after which the process was repeated with additional sections until a total of one million µm^2^ of tissue was collected. The PALM Microlaser System (PALM Microlaser Technologies, Zeiss, Jena, Germany) equipped with the PALM RoboSoftware controlled microscope stage and micromanipulator was used for LCM. PTEC were outlined at the basement membrane using the software and isolated from surrounding tissue by laser ablation prior to being catapulted into a Zeiss AdhesiveCap 500 Opaque collection cap (Zeiss) for subsequent RNA isolation. One cap per “section collection” was used and caps were replaced on their tube and stored on dry ice prior to RNA extraction using the Absolutely RNA Nanoprep kit (Stratagene, Santa Clara, CA, USA) with on-column DNase digestion. Confirming PTEC purity, initial quantitative RT-PCR optimization experiments failed to amplify non-PTEC expressed genes (aquaporin-2 and tamm-horsfall protein) from three million µm^2^ LCM isolated PTEC (data not shown).

### Multiplex Tandem-PCR

This methodology uses two-step MT-PCR gene-disc technology developed in commercial collaboration with AusDiagnostics Pty Ltd (Sydney, Australia). In the first step the RNA from a single one million µm^2^ PTEC extraction was converted to cDNA and amplified in a multiplex single tube reaction for 25 cycles using AusDiagnostics gene-specific primers and master mix reagents. The product was then diluted 1/200 and dispensed into duplicate individual tubes containing specific nested primers for each gene of interest and amplified using real-time PCR for a further 45 cycles with a SYBR Green master mix in a Rotor-Gene Q thermal cycler (QIAGEN, Hilden, Germany). Genes were “called” by AusDiagnostics Software protocols that analyze products based upon melt temperature, purity and quantity values. All “called” results were manually verified during data analysis. Expression levels were normalised to the internal house-keeping genes GAPDH and BTF3 which were assigned an arbitrary value of 1000. Primer and product sequences are not reported due to commercial in-confidence agreements with AusDiagnostics Pty Ltd. We analyzed a total of 30 genes in duplicate for every sample. Genes are listed in Table 1.

**Table 1:** MT-PCR genes examined and the associated disease pathway.

	Gene Symbol	Official Name
House Keeping	GAPDH	glyceraldehyde-3-phosphate dehydrogenase
	BTF3	basic transcription factor 3
Fibrosis	TGFB1	transforming growth factor, beta 1
	SERPINE1	serpin peptidase inhibitor, clade E, member 1
	SMAD3	SMAD family member 3
	COL3A1	collagen, type III, alpha 1
	COL1A2	collagen, type I, alpha 2
	P4HA2	proline 4-hydroxylase, alpha polypeptide II
	SERPINH	serpin peptidase inhibitor, clade H (heat shock protein 47), member 1
	THBS1	thrombospondin 1
Inflammation	TNF	tumor necrosis factor (TNF superfamily, member 2)
	IL6	interleukin 6 (interferon, beta 2)
	IL8	interleukin 8
	NFKB1	nuclear factor of kappa light polypeptide gene enhancer in B-cells 1
	CCL2	chemokine (C-C motif) ligand 2/monocyte chemotactic protein 1
	C3	complement component 3
	BMP7	bone morphogenetic protein 7 (osteogenic protein 1)
	BMP6	morphogenetic protein 6
	Kim-1	kidney injury molecule-1/hepatitis A virus cellular receptor 1
	SPP1	secreted phosphoprotein 1 (osteopontin, early T-lymphocyte activation 1)
EMT	VIM	vimentin
	ACTA2	actin, alpha 2, smooth muscle, aorta
	TJP1	tight junction protein 1 (zona occludens 1)
	S100A4	S100 calcium binding protein A4
	TWIST1	twist homolog 1
	SNAI1	snail homolog 1
Apoptosis	BCL2	B-cell CLL/lymphoma 2
	BAD	BCL2-antagonist of cell death
	BAX	BCL2-associated X protein
	CASP3	caspase 3, apoptosis-related cysteine peptidase
	CASP8	caspase 8, apoptosis-related cysteine peptidase
	FAS	Fas (TNF receptor superfamily, member 6)

### Quantitative RT-PCR

Real-time PCR (RT-PCR) primers were designed and supplied by AusDiagnostics Pty Ltd. Three million µm^2^ of PTEC were extracted by LCM from biopsies and total RNA was isolated from cells with the Absolutely RNA Nanoprep Kit (Stratagene) according to the manufacturer's instructions. Reverse transcription was performed using random hexamers (Invitrogen, Grand Island, NY, USA) and Superscript III Reverse Transcriptase (Invitrogen). RT-PCR reactions were performed using RT^2^ SYBR Green qPCR master mix (QIAGEN). GAPDH was used for normalization of cDNA input, and RT-PCR reactions were performed using a Rotor-Gene Q thermal cycler (QIAGEN) according to the manufacturer's instructions: initial denaturation at 95°C for 10 min, followed by 45–50 cycles at 95°C for 15 sec and at 60°C for 60 sec. Data analysis was performed using Rotorgene software (QIAGEN) and the delta delta Ct (ΔΔCt) method.

### Immunohistochemistry

6 µm cryosections were prepared and dried overnight prior to fixation in acetone/ethanol. Sections were blocked for 30 min in 4% milk and 15 min in 10% goat serum and incubated with primary Ab diluted in Tris-buffered saline (TBS) for 1 hour at room temperature. Primary Abs were mouse anti-human Kim-1 (Clone 219211; R&D Systems, Minneapolis, MN, USA), mouse anti-human Collagen III (Clone FH-7A; Abcam, Hong Kong) and mouse anti-human Vimentin (Clone V9; Dako, Glostrup, Denmark). Following three 5 min washes in TBS, sections were treated with 1% H_2_O_2_ in TBS for 10 min to block endogenous peroxidase prior to 30 min incubation in goat anti-mouse horseradish peroxidase. Sections were then washed 3 times in TBS and developed with DAB for 5 min prior to light counterstaining with haematoxylin and eosin.

### Statistical Analysis

Due to the skewed and wide distribution of the data, gene expression values were log transformed, which enabled the application of parametric statistics. As the log of zero is undefined, one was added to all measurements in order to preserve the zeros in the derived data set. Multiple comparisons of gene expression values for disease biopsies (stratified on histopathology, renal function, proteinuria and clinical diagnosis) against healthy controls were performed using a one-way ANOVA with Dunnett's post-test. Statistical tests were performed using Prism 5.0 analysis software (GraphPad Software, La Jolla, CA, USA). P values ≤0.05 were considered statistically significant.

## Results

### Multiplex Tandem-PCR – PTEC gene expression levels correlate with histology, renal function and proteinuria

We initially compared the means of the disease group to the control group for all 30 genes in the panel. A total of 9 genes discriminated disease samples from controls: Kim-1, C3, P4HA2, THBS1, COL3A1, ACTA2, TJP1, VIM and FAS (Table 2). For example, the expression of Kim-1 was, on average, 1.93 units higher in the disease group as compared to the control group and P4HA2 was, on average, 1.91 units lower in the disease group as compared to the control group.

**Table 2:** The mean log relative gene expression (normalised to GAPDH) of nine genes able to differentiate disease from healthy control biopsies.

	Mean Control	Mean Disease	Disease-Control	p-value
***Kim-1***	0.24	2.17	1.93	<0.001
***P4HA2***	6.24	4.33	−1.91	0.002
***C3***	0.55	2.15	1.6	0.024
THBS1	8.37	6.45	−1.93	0.038
COL3A1	0.43	4.20	3.76	<0.001
ACTA2	5.88	3.83	−2.05	0.012
TJP1	2.40	3.86	1.46	0.033
VIM	0.22	4.34	4.12	<0.001
FAS	4.17	6.32	2.16	0.005

Kim-1, P4HA2 and C3 also demonstrated significant differences within disease groups based on histology (OCI, UCI, IF/TA, ATI and presence of neutrophils/eosinophils), kidney function (eGFR) or proteinuria (uPCR), compared to controls.

We then stratified MT-PCR derived gene transcript data from diseased samples based on: (i) histological criteria (OCI, UCI, IF/TA, ATI and the presence of neutrophils or eosinophils), (ii) kidney function (eGFR) and (iii) degree of proteinuria (uPCR levels). Within these groupings, three genes, Kim-1, C3 and P4HA2 demonstrated significant differences compared to controls.

**Figure 1:**
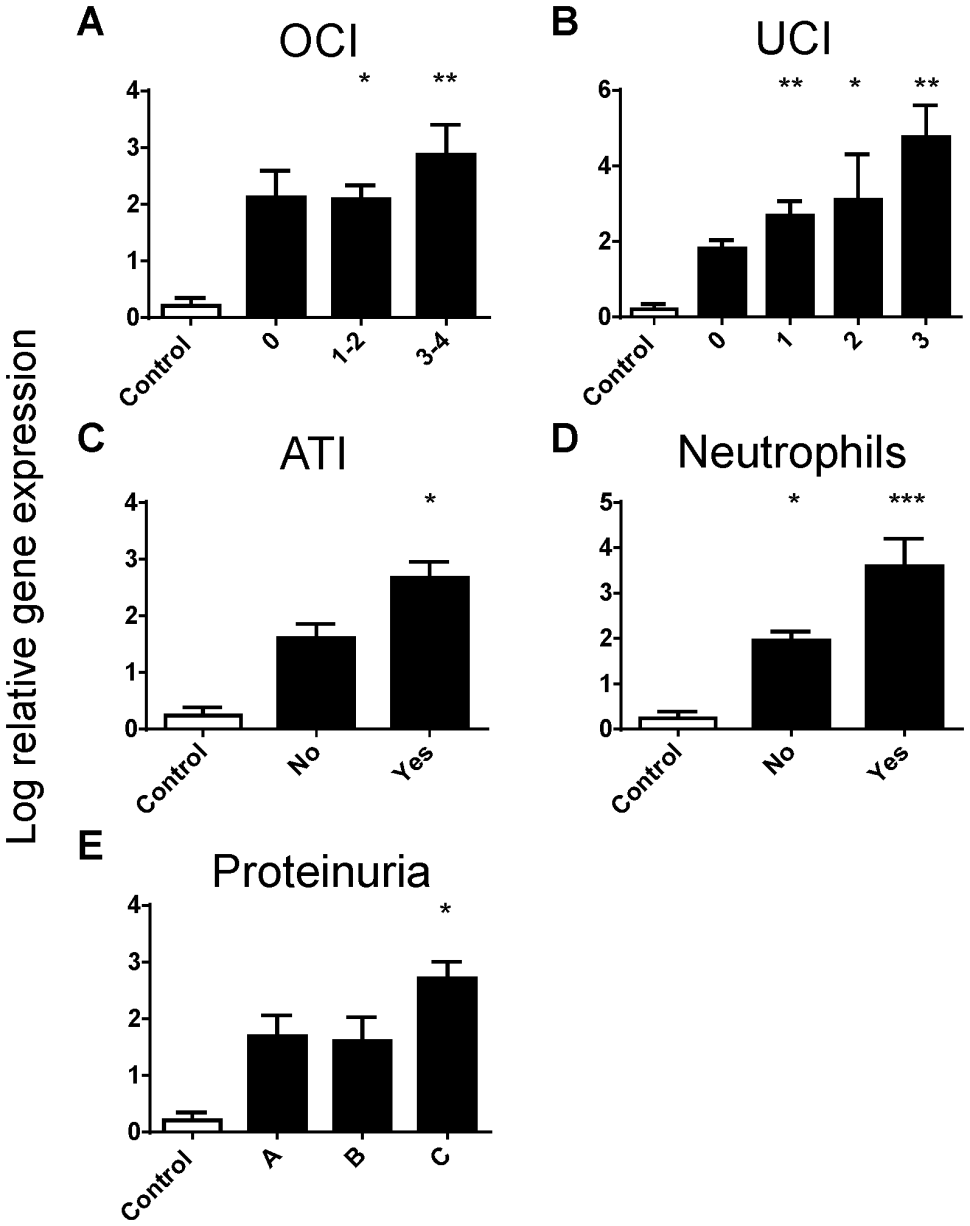
Kim-1 log relative gene expression (normalised to GAPDH) from normal biopsies (Control) and biopsies with (A) overall cortical inflammation (OCI, 0 =  absent, 1–2 = 5–25%, 3–4 = 26–>50%), (B) unscarred cortical inflammation (UCI, 0 = absent, 1 = <5%, 2 = 6–25%, 3 = 26–50%), (C) the presence of acute tubular injury, (D) the presence of neutrophils and (E) proteinuria, defined using a urine protein to creatinine ratio (uPCR) (A = < 100 mg/mmol, B = 100–299 mg/mmol, C = ≥300 mg/mmol). Values are means ± SEM. *P<0.05, **P<0.01, ***P<0.001, one-way ANOVA with Dunnett's post-test.

**Figure 2:**
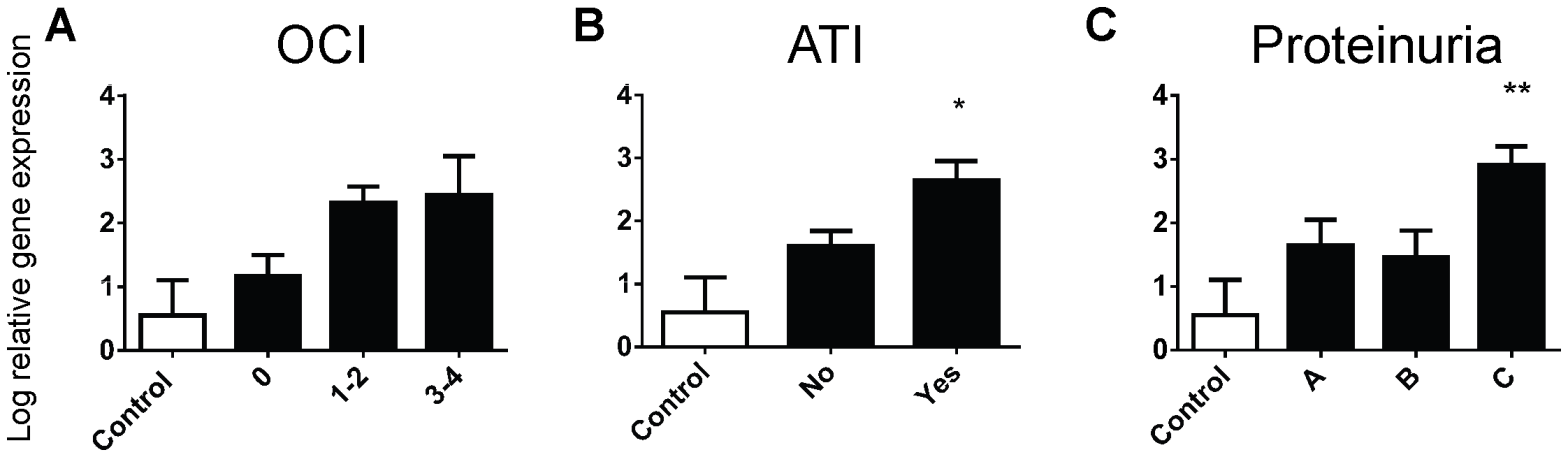
C3 log relative gene expression (normalised to GAPDH) from normal biopsies (Control) and biopsies with (A) overall cortical inflammation (OCI, 0 =  absent, 1–2 = 5–25%, 3–4 = 26–>50%), (B) the presence of acute tubular injury and (C) proteinuria, defined using a urine protein to creatinine ratio (uPCR) (A = <100 mg/mmol, B = 100–299 mg/mmol, C = ≥300 mg/mmol). Values are means ± SEM. *P<0.05, **P<0.01, one-way ANOVA with Dunnett's post-test.

**Figure 3:**
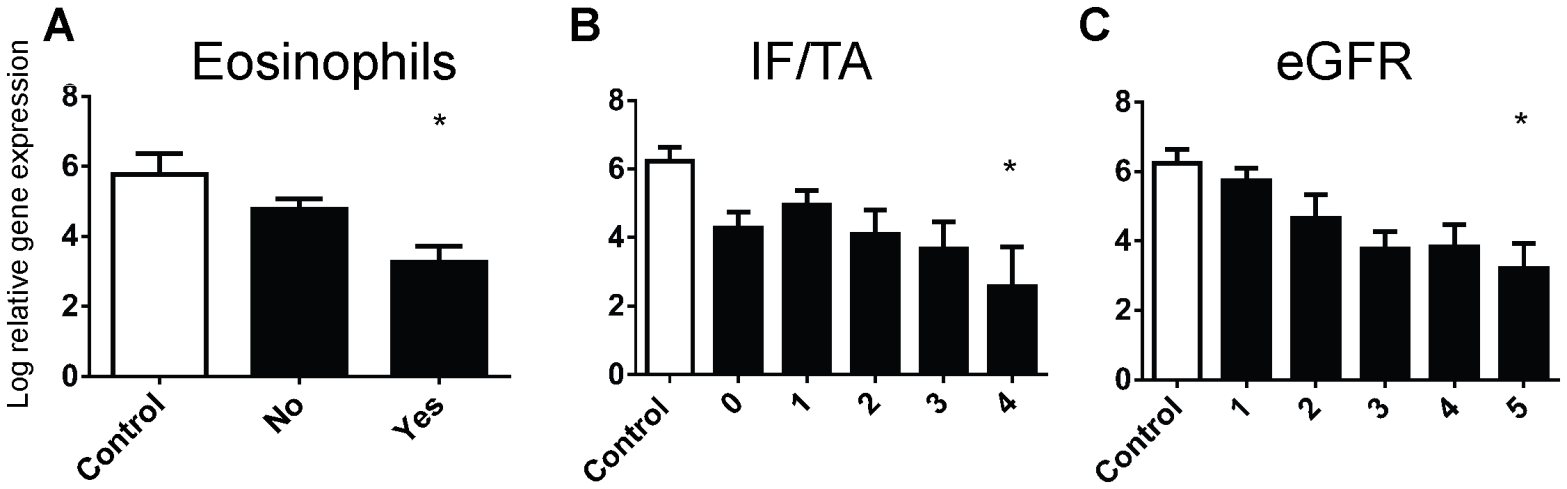
P4HA2 log relative gene expression (normalised to GAPDH) from normal biopsies (Control) and biopsies with (A) the presence of eosinophils, (B) interstitial fibrosis/tubular atrophy (IF/TA, 0 =  absent, 1 = <5%, 2 = 6–25%, 3 = 26–50%, 4 = >50%) and (C) chronic kidney disease (CKD) defined by estimated glomerular filtration rate (eGFR, 1 = ≥90 ml/min/1.73 m^2^, 2 = 60–89 ml/min/1.73 m^2^, 3 = 30–59 ml/min/1.73 m^2^, 4 = 15–29 ml/min/1.73 m^2^, 5 = <15 ml/min/1.73 m^2^). Values are means ± SEM. *P<0.05, one-way ANOVA with Dunnett's post-test.

Levels of Kim-1 significantly correlated with increasing OCI (Fig 1a), and Kim-1 expression was significantly elevated in all disease groupings with UCI (Fig 1b). In line with the literature, Kim-1 expression was significantly higher in biopsies with ATI (Fig 1c) and infiltrating neutrophils (Fig 1d), histological markers of acute kidney disease. Biopsies from patients with the heaviest proteinuria, a marker for progression of uncontrolled kidney disease, also demonstrated significantly elevated levels of Kim-1 expression (Fig 1e). C3 expression levels correlated positively with OCI when compared to no inflammation and controls (Fig 2a). Furthermore, a significant increase in C3 expression was demonstrated in biopsies from patients with acute tubular injury (Fig 2b) and those with the heaviest proteinuria (Fig 2c). In contrast, P4HA2 expression was significantly lower in diseased biopsies with eosinophils (Fig 3a), whilst a trend for decreasing expression of P4HA2 with increasing levels of IF/TA (Fig 3b) and decreasing kidney function (eGFR) (Fig 3c) was observed.

### Multiplex Tandem-PCR – PTEC gene expression levels correlate with clinical diagnosis

**Figure 4:**
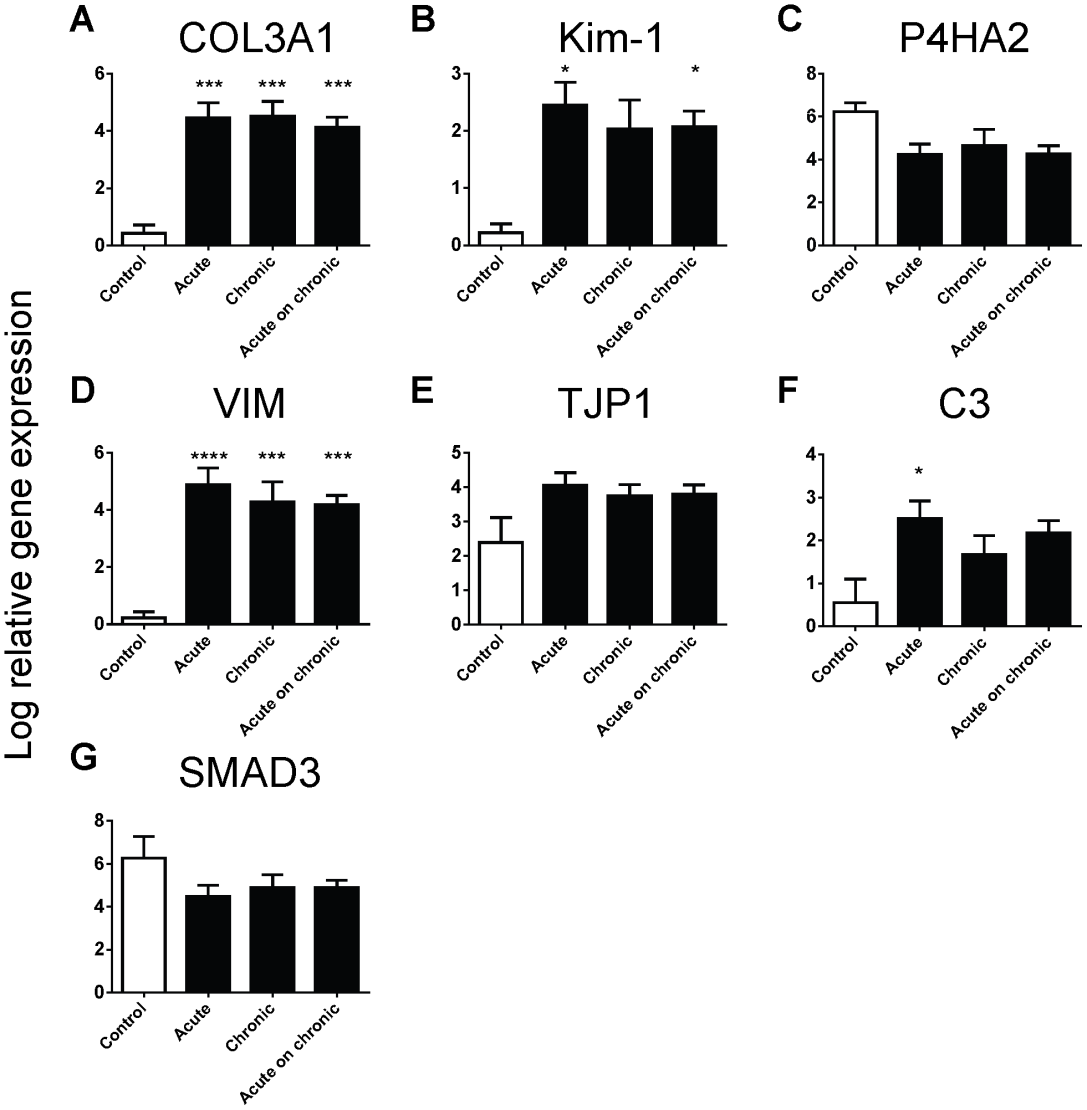
The log relative gene expression (normalised to GAPDH) of COL3A1, Kim-1, P4HA2, VIM, TJP1, C3 and SMAD3 across clinical acute, chronic and acute-on-chronic diagnoses. Values are means ± SEM. *P<0.05, ***P<0.001, ****P<0.0001, one-way ANOVA with Dunnett's post-test.

When we stratified gene results against clinical acute, chronic and acute-on-chronic groups, we found increased expression compared to controls of Kim-1, C3, COL3A1, VIM, SMAD3 and TJP1 across all clinical groupings and decreased expression of P4HA2 compared to controls across all clinical groupings (Fig 4). However there were no significant differences of gene expression patterns between groups for each individual gene.

**Figure 5:**
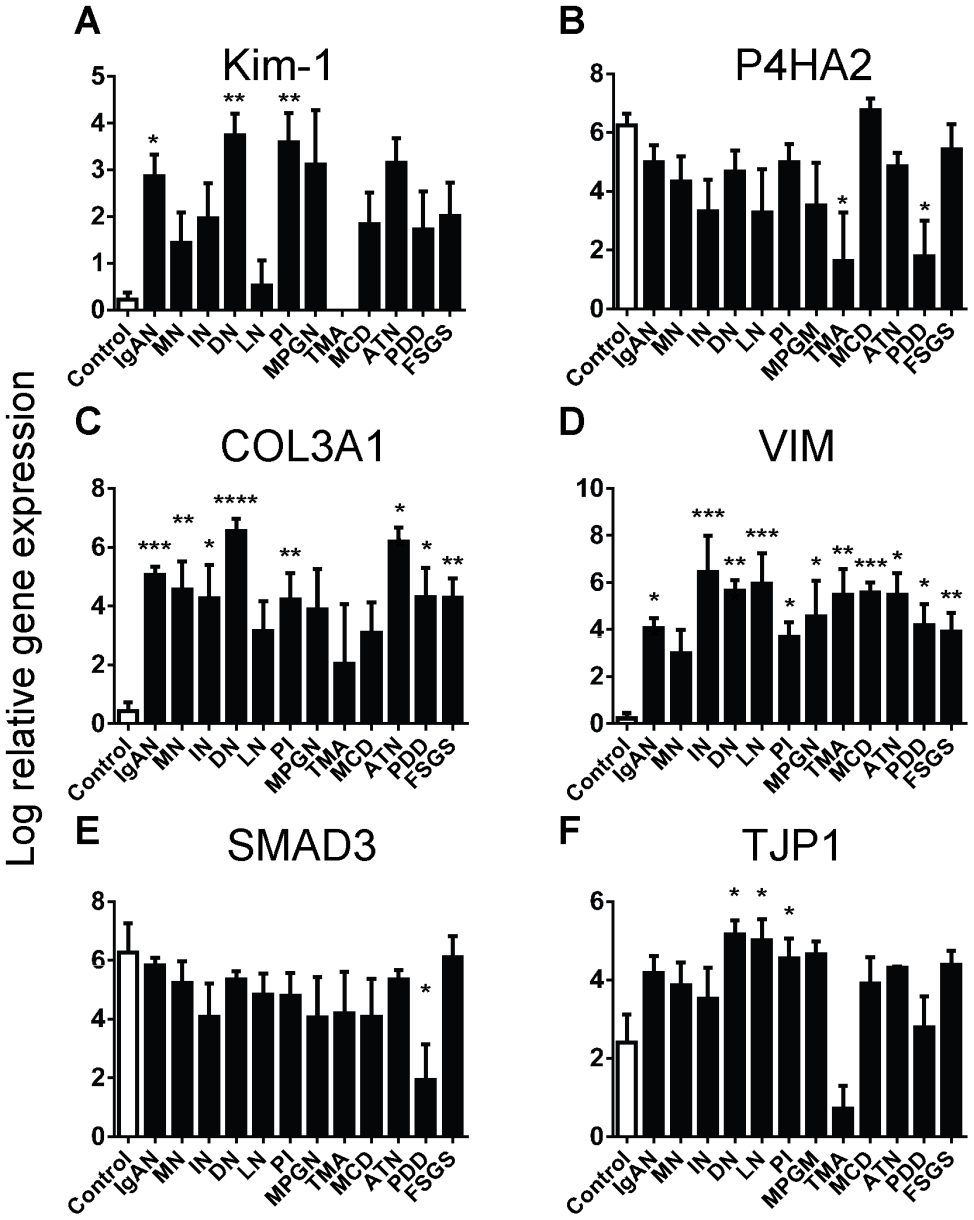
The log relative gene expression (normalised to GAPDH) of Kim-1, P4HA2, COL3A1, VIM, SMAD3 and TJP1 across disease diagnoses. Values are means ± SEM. *P<0.05, **P<0.01, ***P<0.001, ****P<0.0001, one-way ANOVA with Dunnett's post-test.

When we compared gene results against biopsies grouped into disease diagnoses, Kim-1, P4HA2, COL3A1, VIM, SMAD3 and TJP1 were consistently expressed at different levels to control biopsies across a number of primary diagnoses (Fig 5). The mean expression of Kim-1 was higher than controls in biopsies from all disease groups except TMA, and significantly higher than controls in patients with IgAN, DN and PI (Fig 5a). The mean expression of P4HA2 was lower than controls in biopsies from all disease groups except MCD and significantly lower in patients with TMA and PDD (Fig 5b). The mean expression of COL3A1 was higher than controls in biopsies from all disease groups, and significantly higher than controls in patients with IgAN, MN, IN, DN, PI, ATN, PDD and FSGS (Fig 5c). The mean expression of VIM was higher than controls in biopsies from all disease groups and significantly higher than controls in all groups except MN (Fig 5d). The mean expression of SMAD3 was lower than controls in biopsies from all disease groups and significantly lower in biopsies from PDD patients (Fig 5e). The mean expression of TJP1 was higher than controls in biopsies from all disease groups except TMA and significantly higher than controls in patients with DN, LN and PI (Fig 5f).

### Confirmation of MT-PCR results at the molecular and protein level

**Figure 6:**
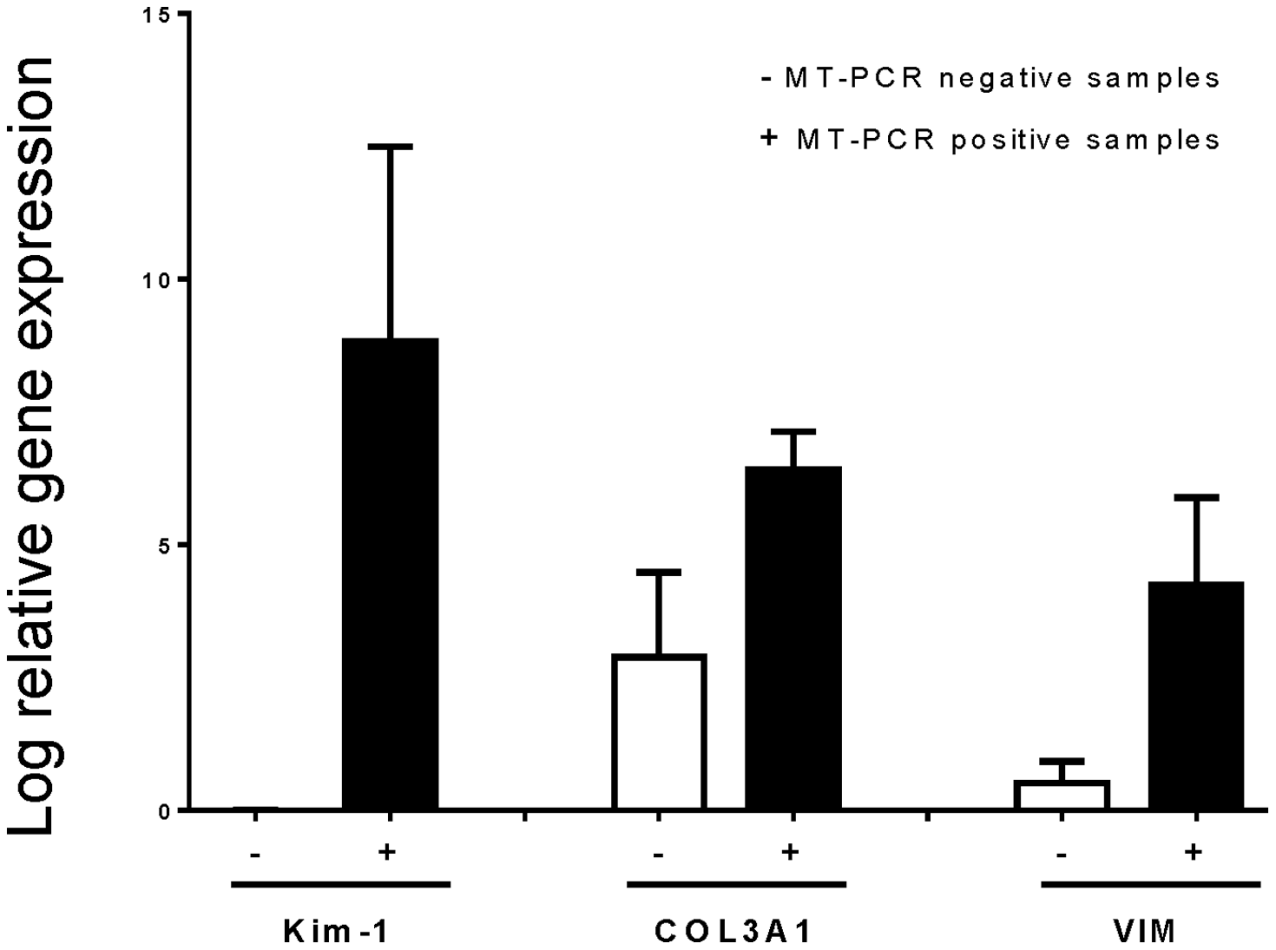
The log relative gene expression (normalised to GAPDH) of Kim-1, COL3A1 and VIM defined by real-time PCR from 3 biopsies with low/no MT-PCR expression (−) and 3 biopsies with medium/high MT-PCR expression (+) for that gene. Values are means ± SEM.

**Figure 7:**
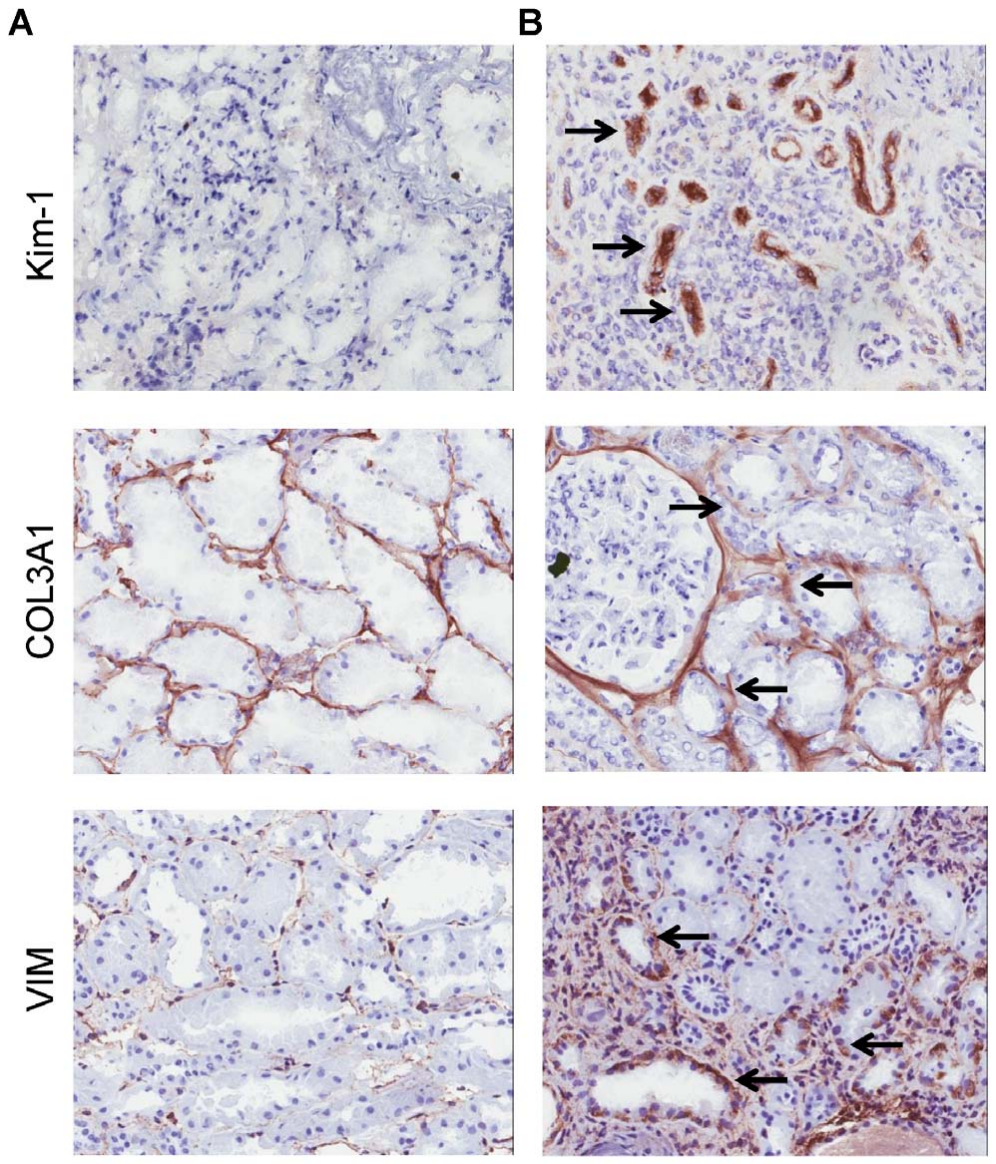
Immunohistological localization of Kim-1, COL3A1 and VIM in frozen kidney sections undertaken on (A) biopsy negative for that gene by MT-PCR and (B) biopsy positive for that gene by MT-PCR. Arrows indicate positive staining within PTEC. A representative result from three donor experiments are shown (mag ×100). There is evidence of Kim-1 staining from the protainaceous material within the lumen of some proximal tubules and COL3A1 staining is evident both within PTEC and in the interstitium, adjacent to the basement membrane.

To confirm our MT-PCR results at the molecular level, we undertook standard quantitative RT-PCR analysis of three candidate genes from our MT-PCR screening panel. We analyzed three different patient biopsies that had high expression of the relevant gene by MT-PCR and three different patient biopsies which had low/negative expression of the relevant gene by MT-PCR. We consistently detected greater levels of Kim-1, COL3A1 and VIM from biopsies with high expression of the respective genes compared to biopsies with no/low gene expression (Fig 6). We performed immununohistochemical analysis of these three gene products to correlate gene transcription levels to protein expression in tissue. We confirmed strong staining for Kim-1, COL3A1 and VIM in PTEC of biopsies that expressed high transcript levels by MT-PCR, with low or negative staining patterns in biopsies that had negative or very low transcript expression levels by MT-PCR (Fig 7). Extracellular staining of Kim-1 in the proteinacious material of the proximal tubule and of COL3A1 in the interstitium, adjacent to the basement membrane, was also noted.

## Discussion

PTEC are believed to play a central role in the progression of many different types of kidney disease, irrespective of whether the initiating aetiology occurs in the glomerulus or the interstitium. Various insults including increased protein, elevated glucose, AGE-modified proteins, oxidative stress, toxins, drugs and cytokines from infiltrating inflammatory cells can all impact on PTEC and perturb their normal physiology. The functional consequences of this perturbed function including aberrant chemokine secretion, EMT and cell death have primarily been studied in cell culture and animal model systems. Using the novel techniques of LCM and MT-PCR we have been able to demonstrate for the first time that PTEC purified from diseased human kidney demonstrate altered gene transcription profiles representing multiple pathobiological pathways that associate with markers of the clinical phenotypes of both acute and chronic kidney disease.

**Kim-1** is a phosphatidylserine receptor that recognises stress and danger signals, like oxidized lipoproteins and apoptotic cells, and directs them to lysosomes [26]. Kim-1 gene expression appears quiescent under normal physiological conditions in the kidney but is rapidly up-regulated and transcribed into protein following injury. Kim-1 then localizes at very high levels on the apical membrane of PTEC where its extracellular domain is susceptible to metalloproteinase-mediated Kim-1 cleavage [26]. This soluble Kim-1, excreted in the urine, is a recognized acute kidney injury biomarker. Our findings that PTEC expression of Kim-1 is up-regulated in biopsies with acute tubular injury compared to both control and disease biopsies with no acute tubular injury is in line with the established literature. Not unexpectedly, high Kim-1 levels in PTEC are also associated with the presence of neutrophils, a cell of acute inflammation, in the interstitium.

Our findings also describe a relationship between Kim-1 expression in PTEC and the histological severity of kidney inflammation, renal function and amount of proteinuria. In addition, we report significantly elevated levels of Kim-1 in biopsies from patients diagnosed with IgA, DN and PI compared to controls. A review of the literature reveals elevated levels of Kim-1 in a number of kidney diseases. Kwon *et al* recently demonstrated that Kim-1 expression predicts renal outcomes in IgA nephropathy [27], whilst elevated Kim-1 levels have also been reported in DN [28] and decreasing levels associated with regression of microalbuminuria in type 1 diabetes, suggesting that tubular dysfunction is a critical component of the early course of DN [29]. This corroboration of our findings with the established literature is proof of concept of the validity of our LCM/MT-PCR approach to identifying PTEC specific genes involved in the pathobiology of kidney disease.

**C3** is pivotal in the activation of the complement system and its processing by C3 convertase to C3a and C3b is the central reaction in both the classical and alternative complement pathways. C3 is a known inflammatory mediator, with C3a increasing vascular permeability and C3b promoting inflammatory cell interactions. PTEC production of C3 gene transcripts and protein has been reported in both in-vitro and in-vivo models [30], [31] and Zoja *et al* [31] reported the co-localisation of interstitial inflammatory infiltrates with C3 expressing proximal tubules. Welsh *et al* also reported focal PTEC expression of C3 was always associated with localised interstitial infiltrate or focal tubular atrophy [32]. This is supported in our findings where there is a strong trend for increased C3 expression in disease biopsies with OCI and a significant positive relationship between expression of C3 and acute tubular injury. Our results support a therapeutic strategy akin to antibiotics; that is, time dependent therapeutic inhibitory targeting of C3 whilst the inflammatory process is active in the pathogenesis, possibly with proteinuria as a surrogate, and not during other pathological processes.

**P4HA2** catalyses the post-translational formation of 4-hydroxyproline in -Xaa-Pro-Gly- sequences in collagens and other proteins and is essential to the proper three-dimensional folding of newly synthesized procollagen chains. To our knowledge, no other groups have studied P4HA2 transcription in human PTEC and only Rastaldi et al [33] have reported on the apparent up-regulation of its protein in diseased biopsies. The significance of decreased expressions levels of this gene in PTEC from biopsies with moderate to severe CKD and IF/TA is therefore counterintuitive. However, prolyl hydroxylation via P4HA2 is part of the pathway by which hypoxia inducible factor-1 (HIF-1) is degraded. HIF-1 is known to regulate many important genes involved in angiogenesis, glucose utilization, proliferation and survival including erythropoietin, vascular endothelial growth factor and hemeoxygenase-1 [34]. It could be hypothesized that a decrease in PTEC expression of P4HA2 would promote the expression and activity of HIF-1, resulting in the expression of many genes required for survival within the compromised kidney environment of CKD. Therefore, our findings that decreased levels of P4HA2 correlate with reduced kidney function provide a new finding that warrants further investigation.

**COL3A1** participates in the fibrotic process in the renal tubulo-interstitium, where the abnormal extracellular matrix (ECM) in fibrosis consists of an excess of normal components, such as collagen type IV, and an accumulation of proteins that are absent in the normal ECM, such as collagen type I and type III [35]. Although most collagen type III is reported to be produced by myofibroblasts in the kidney, it has also been demonstrated to be produced by PTEC cell lines *in vitro* [36] and by PTEC in diseased kidney biopsies [33]. The finding here that PTEC also produce COL3A1 *in vivo*, across a range of disease aetiologies, adds novel findings to the PTEC literature.

**VIM** is the characteristic cytoskeletal protein of mesenchymal cells and its expression in epithelial cells is a de-differentiation marker representing an early step in EMT. In their immunohistochemical studies on EMT in human biopsies, Rastaldi *et al* were unable to detect any vimentin positive tubules in healthy biopsies but reported vimentin positive PTEC from most disease biopsies studied [33]. We hypothesize that the increased PTEC expression of VIM transcripts in our disease biopsies reflects an early pathological response because we rarely detected increased ACTA2, a second, and later, marker of EMT, within PTEC. Galichon and Hertig came to a similar conclusion, reporting that vimentin is an early and reversible marker of EMT in kidney disease [37]. Thus, transcription of vimentin may have potential as a kidney disease biomarker at early and potentially reversible stages. Our observations that ACTA2 itself did not correlate with fibrosis scores or other markers of pathology was somewhat surprising to us as the product of this gene has been implicated in the fibrotic process, particularly in animal models [38]. However, others such as Rastaldi *et al* [33] have also reported minimal increases of smooth muscle actin in PTEC from human fibrotic biopsies.

**SMAD3** is a member of the receptor-activated Smads (R-Smads) which are central mediators of TGF-β signaling. Induction and activation of the profibrotic cytokine TGF-β in the kidney, either alone or in combination with other cytokines such as epidermal growth factor, fibroblast growth factor-2 or angiotensin II, induces kidney damage through a range of pathobiological processes, including EMT, apoptosis and fibrosis (reviewed [39]). SMAD3 is also involved in other non-redundant signaling pathways and biological functions (reviewed [40]). We were quite surprised to note that all of our disease biopsies had lower SMAD3 levels than our control biopsies. However the literature reports that TGF-β can down-modulate SMAD3 gene expression in human glomerular mesangial cells over the short term (24 hours) and in renal tubular epithelial cells over the longer term (5–7 days) [41]. Therefore, our group hypothesize, for the first time, that the transcriptional inhibition of SMAD3 may form part of a biological control loop for TGF-β within human PTEC.

**TJP1** or ZO-1 is a scaffolding protein that interacts with multiple other proteins to form and maintain tight junctions between cells. Levels of TJP1 are relatively low in proximal tubules compared to distal tubules (reviewed by [42]) reflecting their greater paracellular transport functions. As proximal tubule cells become fully differentiated they express more TJP1 [43]. This raises the intriguing possibility that our observed increases in TJP1 transcription in most disease states is a counter-regulatory response to the vimentin up-regulation induced early stages of PTEC EMT– counter acting forces in the de-differentiation:differentiation of PTEC homeostasis.

Collectively our study demonstrates that the technologies of LCM coupled with MT-PCR are feasible in the clinical setting where levels of PTEC RNA are available only in picogram amounts. Despite the incredible complexity of PTEC transcriptional profiles, their products and interactions, these technologies provide simultaneous experimental observations of multiple molecules of interest, informing better targeting of therapies and potential new renal biomarkers. However, our findings do not, at this stage, define a PTEC consensus chronic kidney disease transcript signature, aligning with the concept that chronic disease represents the cumulative dynamics of transient, repetitive or persistent acute insults varying over time.

Although we have used MT-PCR to examine genes involved in kidney pathobiology, the technique can be modified to examine any genes or gene pathways of interest. For instance, we have recently reported that human PTEC are able to modulate autologous immune responses [44], [45] and we are currently modifying our protocols to examine the expression of immune mediator genes in PTEC derived from inflammatory disease biopsies.

In conclusion, using novel methods that give unprecedented access to real time human kidney tissue, we report alterations in PTEC gene and protein expression, representing multiple pathobiological pathways that associate with markers of the clinical phenotypes of both acute and chronic kidney disease. Where the amount of starting RNA is small, MT-PCR has the power to simultaneously observe multiple molecules representing several signaling pathways at the level of a single cell population. We found the diversity of pathways implicated in kidney disease include inflammatory, complement, collagen folding, fibrosis, cytoskeletal and cell scaffolding pathways, ascribing PTEC a pivotal role in regulatory networks of kidney homeostasis in health and disease.
